# Using residents and experts to evaluate the validity of areal wombling for detecting social boundaries: A small-scale feasibility study

**DOI:** 10.1371/journal.pone.0305774

**Published:** 2024-08-26

**Authors:** Meng Le Zhang, Aneta Piekut, Zanib Rasool, Lydia Warden, Henry Staples, Gwilym Pryce

**Affiliations:** 1 Department of Economics, University of Sheffield, Sheffield, United Kingdom; 2 Sheffield Methods Institute, University of Sheffield, Sheffield, United Kingdom; 3 Rotherham United Community Sports Trust, Rotherham, United Kingdom; King Fahd University of Petroleum & Minerals, SAUDI ARABIA

## Abstract

Several studies have explored the relationship between socially constructed neighbourhood boundaries (henceforth social boundaries) and ethnic tensions. To measure these relationships, studies have used area-level demographic data to predict the location of social boundaries and their characteristics. The most common approach uses areal wombling to locate neighbouring areas with large differences in residential characteristics. Areas with large differences (or higher boundary values) are used as a proxy for well-defined social boundaries. However, to date, the results of these predictions have never been empirically validated. This article presents results from a simple discrete choice experiment designed to test whether the areal wombling approach to boundary detection produces social boundaries that are recognisable to local residents and experts as such. We conducted a small feasibility trial with residents and experts in Rotherham, England. Our results shows that participants were more likely to recognise boundaries with higher boundary values as local community borders. We end with a discussion on the scalability of the design and suggest future improvements.

## Introduction

In this study, we are concerned with the detection of socially constructed neighbourhood boundaries (henceforth social boundaries) between different ethnic and cultural neighbourhoods. There is qualitative evidence from residents about the existence of socially constructed boundaries and neighbourhoods [1–5]. Whilst neighbourhoods and social boundaries may be recognisable to residents, there is ambiguity about their exact boundaries and space which complicates neighbourhood research [6]. Using demographic data, several researchers have proposed different methods for predicting the location of social boundaries. These same studies have sought to establish the relationship between their *predicted* social boundaries and other outcomes. The primary motivation behind this body of research is to explore the relationship between boundaries and conflict, and the contribution this makes to understanding the effects of ethnic or cultural segregation. A key concept is how well-defined social boundaries are: a well-defined boundary indicates an area where there is a sharp and well-recognised divide separating two communities. Fuzzy social boundaries denote locations where the divide between communities is less-recognised and communities may overlap. Some researchers have hypothesised that more conflict and tension occur in fuzzy (i.e. ambiguous) social boundaries due to the threat of out-groups and the potential to contest boundary lines in these areas [7–9]. Others have conjectured that the more clearly the demarcation of social boundaries, the greater the likelihood of conflict due to (i) the symbolic role of such boundaries in evoking territorial behaviour [7, 10, 11], (ii) less mixing [7, 10, 11] and (iii) weakened mechanisms for informal social control [7, 8].

Empirically, Legewie and Schaeffer’s [8] study in New York, USA, revealed more neighbourhood tension (measured in terms of anti-social behaviour) when predicted boundaries were less defined. In contrast Legewie [7] and Dean et al. [10] found that crime rates were higher close to more clearly defined social boundaries (based on violent crime data in Chicago, USA, and data on multiple crime types in Sheffield, UK, respectively). However, Křížková et al.’s [12] study based in Czechia did not find a significant relationship between the location of frontiers and crime. Maguire, French, and O’Reilly [13] did not use any boundary-detection algorithm but found a correlation between ‘peace lines’ and poorer mental health in Northern Ireland (higher depression and anxiety).

Despite this burgeoning body of literature, no studies to date have sought to test whether *predicted* social boundaries (that is, those identified through demographic and/or geographical data) correspond to social boundaries as recognised by residents. Our review of the literature on social boundary detection, including additional follow-up on citations in key papers [7, 8, 10, 14], and contact with authors for further information on related studies or works in progress, is summarised in Table 1. Crucially, we intentionally omitted studies that focused on physical boundaries or other types of discontinuities in space [e.g. edges based on cancer detection, 15], as opposed to socially constructed boundaries.

**Table 1:** Papers detecting social boundaries.

Study	Sample	Method	Variable used to determine boundaries
Legewie and Schaeffer (2016)	New York City, USA	Modified edge detection algorithm adapted from image processing	Proportion of white, black, Hispanic and Asian residents
Legewie (2018)	Chicago, USA	Areal wombling	Proportion of residents from various ethnic backgrounds (based on which ethnic group has the largest difference across two areas)
Neil and Legewie (2023)	New York City, USA	Areal wombling	Proportion of Black and/or Hispanic residents
Dean et al (2019)	Sheffield and Rotherham, UK	Two-step approach Bayesian Areal Wombling	Proportion of Foreign born residents
Křížková et al (2021)	Pardubice, Czechia	Two-step approach Bayesian Areal Wombling	Proportion of Foreign born residents
Kim and Hipp (2021)	Los Angeles County, USA	Difference in characteristics across two sides of a street segement	Proportion of residents from ethnic backgrounds (weighted)
Olner et al (2023)	Rotterdam, Netherlands	Two-step approach Bayesian Areal Wombling	Proportion of ’native’ and ’nonwestern non-native’ residents
Kramer (2017)	Philadelphia, USA	Non-euclidean smoothing to identify sharp changes in demography	Proportion of residents by ethnicity

Among the tools employed for detecting social frontiers, the ‘areal wombling’ method has emerged as perhaps among the most prevalent. This method employs area-level data such as administrative data units or grid squares depending on data availability [16], with boundaries (or edges) connoting the relationship between pairs of geographically contiguous areas. A ‘boundary value’ thereby serves as a statistical measure of the likeness of two neighbouring areas, with *higher* boundary values denoting two areas that are *unalike*. For instance, a high-value boundary (also referred to as a steep boundary, sharp edge, hard edge, or social frontier [8, 10, 14, 17], may consist of two areas with drastically different proportions of non-native residents. While there are differences in how studies construct the boundary value, the simplest (and most common) approach is to use the raw difference in the proportion of ethnic minority or foreign-born residents across two areas. When there are multiple ethnic or cultural groups, different authors elect to use weighted scores or other methods [9, 14]. Some studies also employ statistical modelling to adjust the boundary value for small sample sizes [10, 12, 16].

Though the exact detection method uses varies from study to study, much of this work is ultimately concerned with identifying the kinds of boundaries which will be experienced by ‐ and identifiable to ‐ residents as social boundaries. Some classify statistical boundaries as connoting well-defined social boundaries if they exceed a certain boundary-value threshold [10, 12], whilst others use boundary values directly in their analysis [7, 8, 14]. A key implication of this work is that boundaries between zones that are less alike (i.e. higher boundary values) are associated with well-defined social boundaries and are more easily identified by residents [7, 8, 10, 14, 17], while boundaries with lower boundary values are less likely to be identified as such. However, the lack of validation makes it difficult to establish the robustness of these studies and their findings.

### The current study

This current study uses residents and experts in Rotherham, England to test the validity of an areal wombling approach to detecting social boundaries. We test a key feature of the approach: namely the use of boundary values to detect potential social boundaries. The common assumption is that high boundary values (or steeper boundaries) are associated with well-defined social boundaries [7, 8, 10, 14, 17]. We chose to replicate the boundary detection algorithm outlined in [10] (henceforth referred to as the Bayesian areal wombling). Our boundary values are determined by the difference in the proportion of foreign-born residents in two adjoining areas accounting for uncertainty and spatial autocorrelation [10]. Whilst we do not directly test boundary values calculated using other algorithms, we believe that our results should also apply to studies that use the difference in proportion *without* any statistical adjustment since the the two types of boundary values are highly correlated (spearman’s rank: 0.90).

We conducted a simple discrete choice experiment [18]. We created three maps of the same area with different boundaries using the Bayesian areal wombling approach. Map A contained the boundaries with the highest boundary values whilst map C had the lowest boundary values. Map B contained boundaries that were in between. Participants were then shown pairs of maps and asked which map in each pair best corresponds to local community boundaries. The sequence and order of the maps shown were randomised. Assuming that residents and experts can recognise (but not necessarily recall) social boundaries, we conjecture that participants would choose the map containing borders with higher boundary values. Aside from testing a hypothesis, another motivation behind the study is to explore the feasibility of the method for future replications and follow-on research.

#### Primary objective

Determine whether areal wombling (following [10]) produces boundaries that are recognisable to residents and experts as social boundaries.

#### Hypothesis

We hypothesise that participants will agree with the predictions of the areal wombling algorithm and choose boundaries with higher boundary values.

#### Null hypothesis

Participants are not more or less likely to choose boundaries with higher boundary values.

We found that participants chose the map with the highest boundary value (map A) over other maps. However, there is limited evidence that participants would choose the map with medium steepness over low steepness. Due to implementation issues, we did not achieve the desired sample size set out in our protocol. We discuss limitations and directions for future research.

## Materials and methods

### Study setting

The study was carried out as part of the qualitative strand of an international research project, focused on understanding the dynamics of areas with a high number of well-defined social boundaries. The study was originally designed to be a multi-site trial across six sites in the UK, Norway and Sweden, with urban areas selected based on a higher-than-average degree of segregation, the proportion of foreign-born residents, and the team’s existing research collaborations. Rotherham–a large town in South Yorkshire ‐ was initially selected on this basis. Issues of implementation, particularly the logistical challenges associated with the COVID-19 pandemic which coincided directly with the project, led to Rotherham ultimately being the only case study in which the validation exercise was undertaken. This leads to less statistical power than anticipated in the original protocol (e.g. lower likelihood of rejecting the null hypothesis).

While Rotherham’s population remains predominantly White British (88%), historical migration patterns including a substantial number of arrivals from Pakistan during the 1970s and 1980s, have resulted in significant ethnic diversity in certain areas, with more recent migration from Eastern Europe continuing this trend (see Table 2). Our sample of residents is centred on three neighbourhoods situated in the Rotherham West ward, which align with the Kimberworth Middle Layer Super Output Area (MSOA) and the Masbrough and Bradgate MSOA. Initial conversations with community members identified these areas as having several likely social frontiers.

**Table 2:** Census 2021 demographic data.

Country of birth and ethnicity	England & Wales	Rotherham	Kimberworth (MSOA)	Masbrough and Bradgate (MSOA)
UK-Born	83.2%	93.2%	95.0%	75.3%
Non-UK Born	16.8%	6.8%	5.0%	24.7%
White	81.7%	91.0%	95.2%	64.0%
*White British*	74.4%	88.3%	92.2%	53.9%
*White Other*	7.3%	2.2%	2.4%	7.9%
Asian & British Asian	9.3%	5.3%	2.0%	25.1%
*British Pakistani*	2.7%	3.8%	1.5%	21.1%
Other ethnic groups	10.0%	1.1%	0.3%	5.8%

A study protocol including an analysis plan was created before data collection (Zhang and Piekut, n.d.) and the exercise is based on the 2011 census data (which was the latest census at the time).

### Eligibility criteria

All participants were adults (18+) who were either:

1. Residents who have lived in Rotherham West for at least one year
2. Local ‘experts’ which include councillors, community support officers, local government and housing officers, representatives of community advocacy groups and other third-sector organisations, and various agencies involved in the support and integration of migrant communities in the selected case study area.

### Recruitment

Local experts were recruited for interviews via our local contacts in Rotherham, identified by desk research and by snowball sampling (i.e. further contacts gained from initial interviews).

For residents, we asked each expert to point us to any community web pages, groups or centres which were most suitable to recruit participants. We created a leaflet with information about the research which we distributed via identified channels, including online and offline. Interested participants came back to us via email and telephone.

### Discrete choice experiment

The experiment for this study was conducted either at the beginning or end of a semi-structured interview of approximately 60 minutes in duration. Interview questions pertained to individual experiences of life in their neighbourhood, and perceptions concerning the socio-economic and demographic composition of the wider Rotherham West area. During the interview, a physical map (unrelated to the validation exercise), was also employed to prompt discussion, with participants encouraged to highlight areas inhabited by people of similar and/or distinct identities to themselves. The conversation surrounding the validation exercise, including the selection of maps by each participant, was recorded by the interview team along with the entire semi-structured interview.

**Fig 1:**
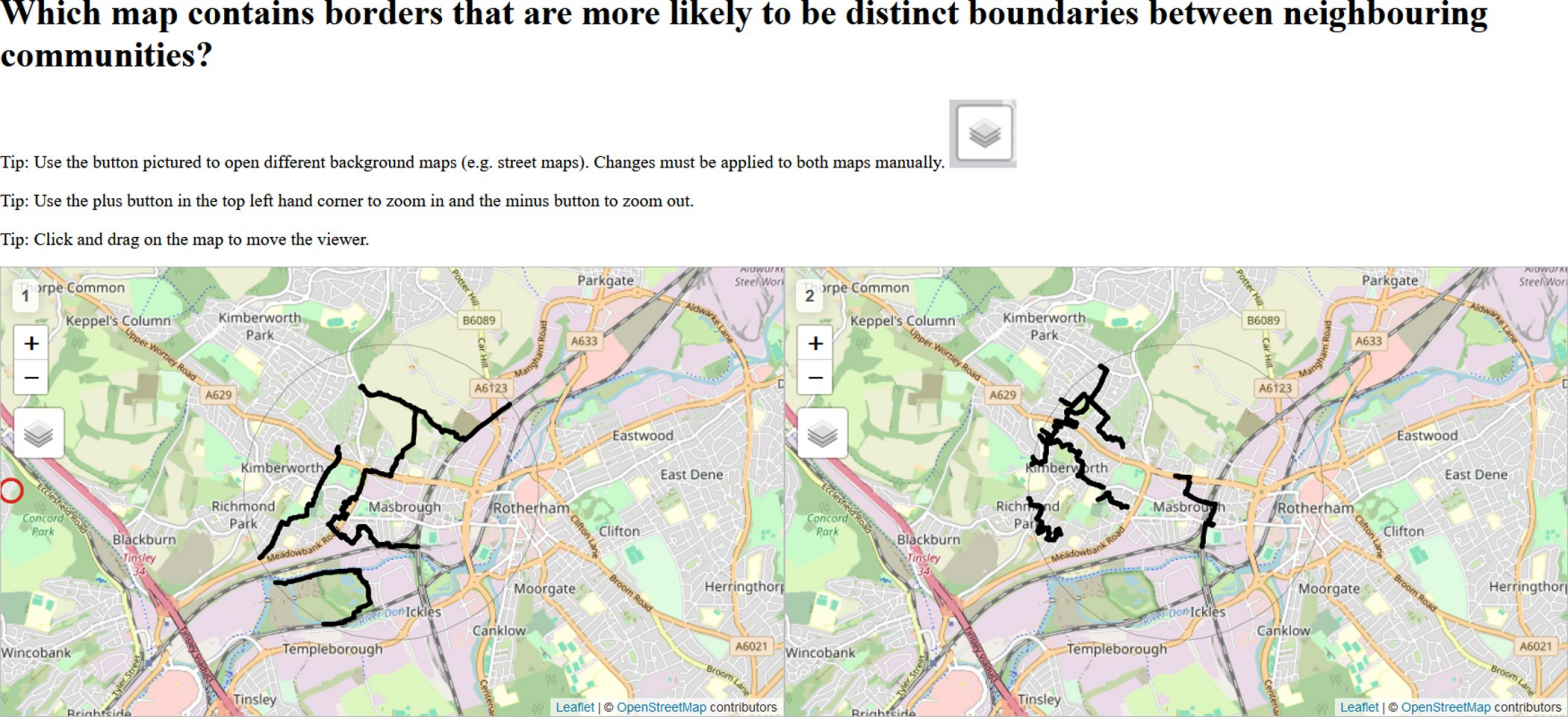
Screenshot of task shown to participants. Both interactive maps are synced. Base map and data from OpenStreetMap and OpenStreetMap Foundation.

The experiment began with a preliminary task, during which participants were provided instructions on how to read and manipulate the interactive pairs of maps. In the case of online interviews, maps were displayed via the ‘share screen’ function, in-person interviews, maps were shown on a laptop or a large external screen, if available. As shown in Fig 1, maps centred on the Rotherham West area, displayed border information within a 1.5km radius, and were synced such that moving the displayed area in the left-hand map had the same effect on the right-hand map, and vice versa. Interviewers verified each participants’ capacity to adjust the maps (scroll, zoom in, zoom out, etc), by requesting them to locate a local landmark. After this preliminary task, participants were shown three pairs of maps (A, B or C) in a randomized sequence and order, and asked to select the map in each pair that, in their view, more accurately represents the social frontiers in the selected area, with particular reference to the migratory background of residents.

For each pair, there is agreement if participants choose the map with higher boundary values (e.g. A over B). Our test statistic is the rate of agreement (with the wombling algorithm): under the null hypothesis, the agreement rate is 50 per cent.

Pilot exercises were conducted before deployment, and the entire validation exercise was expected to take under 10 minutes to complete. We checked that all the maps were similar concerning the number of boundaries and boundary lengths to omit alternative explanations (see S2 File).

#### Intervention

Three maps (A, B and C) were created for use in the exercise. To create the maps, we used the two-step Bayesian areal wombling approach to calculate boundary values based on the difference in the proportion of foreign-born residents (see [10]). Our information comes from the 2011 census and we use residential information for Lower Super Output Areas (LSOA). The average population of LSOAs in England and Wales is 1,614 with 95% of LSOAs having a population of between 1,157 and 2,354. For detecting boundaries, we restrict our data to the Sheffield Travel to Work Area (which contains the town of Rotherham). We denote the boundary value as *Φ* where large values indicate steeper boundaries (i.e. larger differences in proportions of foreign-born residents).

**Fig 2:**
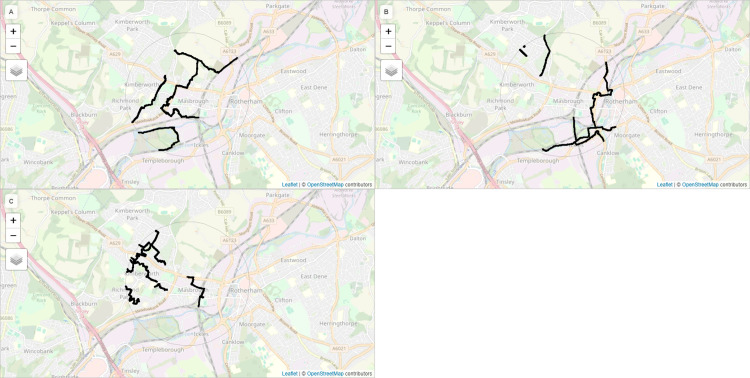
Boundary maps shown to participants in the exercise. Each map is interactive: participants can zoom, drag and change base layers. Base map and data from OpenStreetMap and OpenStreetMap Foundation.

**Fig 3:**
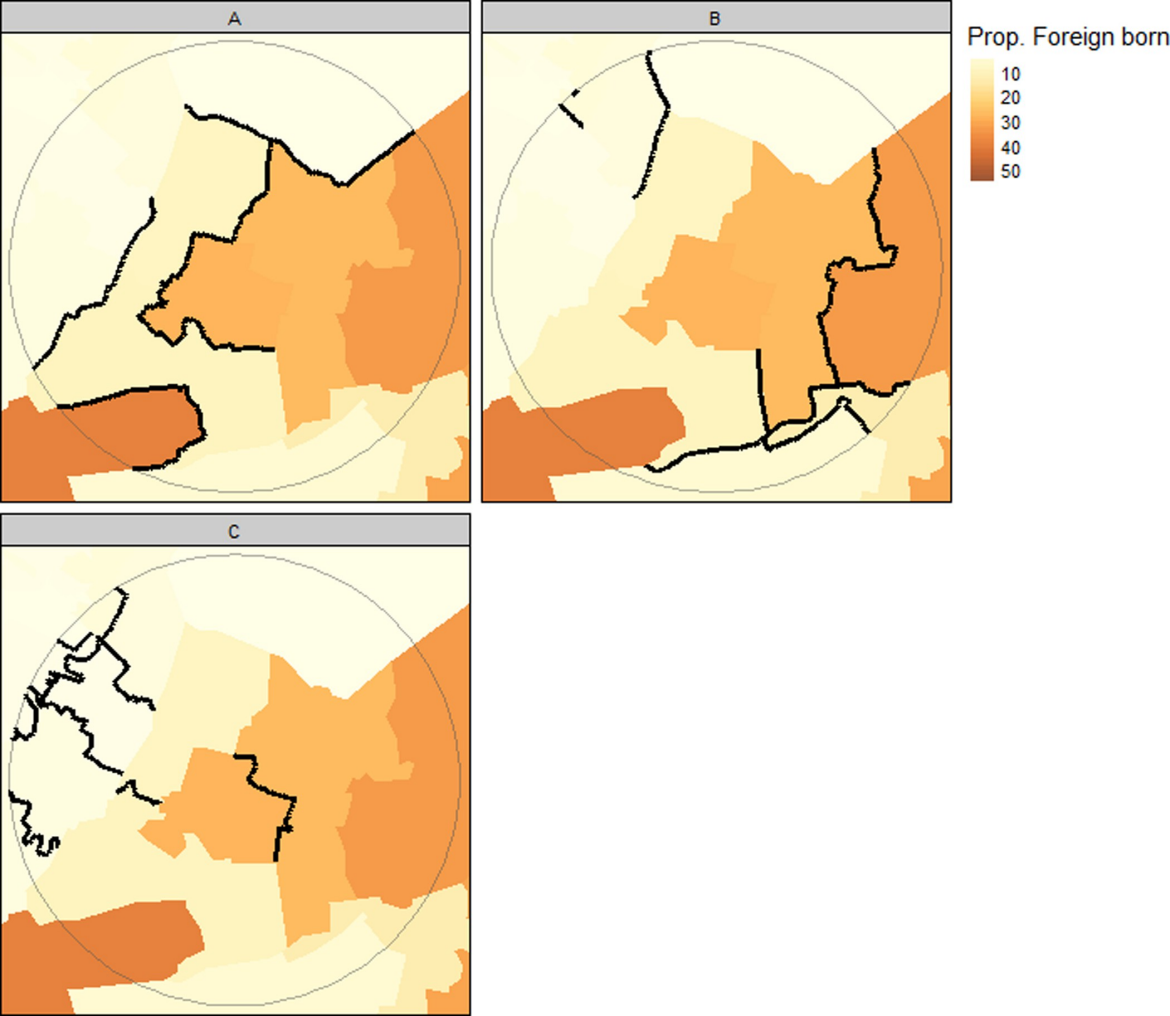
Maps of boundaries in order of boundary values (high to low: A > B > C). Colours represent the proportion of foreign-born residents in each area (not shown to participants).

Then we group boundaries into tertiles and create three maps; where map A contains the steepest set of boundaries (highest tertile), map B contains the middle tertile and Map C contains the lowest tertile. Fig 2 shows the three maps used in the exercise. Fig 3 shows the relationship between boundary sets used in each map and the proportion of foreign-born residents living in different areas.

For the validation exercise, the map pairs shown to participants:

- A and B (pair 1)
- A and C (pair 2)
- B and C (pair 3)

For each pair, we measure the proportion of participants who prefer the map with higher boundary values (i.e. agreement rate). The maps are created in R and exported as leaflet maps within HTML files. The code to recreate the research materials are publically available (see data availability). The data and experiment analysis code is included in S1 File. A more technical explanation of the Bayesian areal wombling routine is contained in S3 File. Participants were shown all three pairs of maps.

*Concealment mechanism and blinding*. The order and sequence were saved onto a file and not shared with the research team responsible for conducting the interviews and accompanying exercise. As such, both the interview team and participants remained unaware of how the maps were designed and generated, as well as their order and sequence during the exercise, until after data collection.

### Sample

Since the exercise was incorporated within a longer qualitative interview, the sample size was determined by the overarching qualitative research design. The final sample consists of 31 participants from Rotherham, England. We are left with 30 participants after eliminating ineligible responses (i.e. respondents who did not complete the preliminary exercise). Of these, 24 were conducted face-to-face and the remaining six online.

We recorded demographic details for 23 of the participants ‐ the residents (see Table 3). The vast majority of the sample have lived in the Rotherham area for more than 20 years (16 out of 23, e.g. most or all of their lives) with a median age similar to the general Rotherham population (41 years based on the 2021 census). Over half of participants were not ethnically White: the majority of these were British Pakistani. This makes our sample more ethnically diverse than Rotherham (91% white) but roughly similar to the composition in one of the local areas (64% white in Masbrough and Bradgate). With only two exceptions, all of our sample were born in the UK which is broadly representative of Rotherham in general (93.2% born in the UK) but not representative of residents living in Masbrough and Bradgate (75.3%). However, given our low sample size, it is difficult to draw conclusions about representativeness.

**Table 3:** Summary of participant characteristics.

Variable	Value	Freq
Age group	18–29	7
Age group	30–39	3
Age group	40–49	3
Age group	50–59	7
Age group	60 +	2
Ethnic background	Non-white	12
Ethnic background	White	10
Gender	Man	9
Gender	Woman	13
How long lived in the area (bands)	1–9	4
How long lived in the area (bands)	10–19	3
How long lived in the area (bands)	20 +	16

### Statistical analysis

For each map pair, we measure the agreement rate: the proportion of participants who prefer the map with the higher boundary values. We refer to this rate as *P*_*j*_ where *j* denotes map pairing and *N* denotes the number of total responses. The number of responses that preferred the map with the steeper boundary is *NP*_*j*_. We use the binomial distribution to calculate p-values: under the null hypothesis, *NP*_*j*_ is binomially distributed with a probability equal to 0.5. We can also calculate the overall agreement rate across all map pairs. All participants responded to all map pairs.

We conducted additional tests for sequencing, ordering, and better agreement over time. For sequencing effects, we use a Fischer’s exact test to check for an association between agreement (agreed/ disagreed) and when a map pair were shown (first/ second/ third). Sequencing tests for whether participant engagement wanes during the exercises due to distractions or boredom, thereby reducing the agreement rate. If engagement does not wane, then the sequence in which pairs are shown should not affect agreement (i.e. the first pair will not have a lower or higher agreement rate than the last pair). For ordering, we use a Fisher’s exact test to check for an association between agreement (agreed/ disagreed) and whether the left or right map contained higher boundary values. Ordering tests whether participants are more or less likely to pick the left map in a pair: this may suggest that participants are not fully engaged and want to complete the exercise quickly. To test whether agreement rates change over time, we split the sample into two halves by chronological order. We use a Fischer’s exact test to check for an association between agreement (agreed/ disagreed) and interview timing (earlier/ later). A higher agreement rate for later participants suggests that the interview team may be nudging participants towards ‘correct’ responses, despite remaining blind to which map displays the higher-value boundary. Since the order of participation in the experiment was not random, with experts generally having been approached earlier in the qualitative research process, this test may also reflect changes in levels of engagement. All analyses were conducted in the software R [19] and code is included in S1 File (with outputs in S2 File). For testing, p < 0.05 is used as the threshold for statistical significance.

### Ethics and consent

This study was approved by the University of Sheffield ethics committee (application number 042378). Written consent was obtained from participants to use their data.

We sent participant information sheets well before the interviews (either via email or traditional post), so participants had time to read them and ask questions. Consent for online interviews with experts was provided by ticking a box on a Google Form. Following the interview, each participant was provided a £25 shopping voucher in recognition of their time and shared knowledge.

### Project timelines

Recruitment of participants for the qualitative interviews began in late 2021. The validation exercise design and materials were created between June and August 2021. Ethical approval from the University of Sheffield was secured on 30th July 2021. The interviews for Rotherham took place between September 2021 and the end of September 2022. The analysis of the exercise data began in September 2022.

## Results

The agreement rates for each map pair are: 96.7% (p < 0.001, A vs B), 96.7% (p < 0.001, A vs C) and 60% (p = 0.20, B vs C). There is evidence that the map with the highest boundary value is more likely to better represent well-defined social boundaries. However, we cannot reject the null hypothesis that areas with medium boundary values are more likely to represent social boundaries compared to areas with low boundary values.

Testing the robustness of our study, we do not find any statistically significant sequencing effects (fisher exact test, p = 0.667); ordering effects (fisher exact test, p = 0.570); or changes in agreement rate over time (p = 0.384). Our results tables are included in the S2 File (with underlying code in S1 File as well).

## Discussion

To our knowledge, experiments in Urban Studies and Human Geography are rare and this is one of the first trials to empirically test the validity of detection algorithms for social boundaries [also see 20 for a different approach for detecting communities as zones]. The study does have several limitations. The sample size is much smaller than initially anticipated which limits our ability to detect smaller effect sizes.

Despite meeting our primary objective, the study does have a number of limitations. Although we find no major shortfalls in terms of the robustness of the study design, issues were certainly present. The sample size was notably smaller than initially anticipated, limiting our ability to detect smaller effect sizes. Furthermore, although we made efforts to ensure that maps were more or less identical aside from the steepness of the boundaries displayed, we cannot guarantee that participants based their selections purely on the relative values or ‘steepness’ of said boundaries. For instance, many participants remarked that their selections were predicated on boundary lengths: coincidentally, Map A had longer total boundaries (726m) relative to Map B (627m) and Map C (662m). It was originally our intention to mitigate these issues through a multi-site design (which was disrupted by COVID-19). Furthermore, while we did not find significant evidence of inattention, participants on average completed the exercise quicker than anticipated, which may be an indicator of somewhat limited engagement. Relatedly, some accessibility issues were noted during the preliminary phase of the experiment with some participants requesting the interviewer to scroll and zoom on their behalf (“I don’t know how to operate the mouse, I’ll be honest. So–can you do it sorry, I don’t know how to zoom in.”). Lastly, the displayed boundary lines did not always resonate with participants, with some expressing the view that their responses were not based on lived experience but on rough estimation (“l’ll be honest with you, I’m just like guessing.”). Isolation and disruptions to everyday life caused by COVID-19 (e.g. restrictions on travel, working from home) may have also caused deeper divisions between communities and increased the awareness of boundary lines. This may affect the external validity of the study findings but this is difficult to determine from a single study.

It is worth noting that, even if not validated by residents, the boundaries produced through areal wombling may be valid for other purposes. This is significant, as data collection was arguably the biggest challenge underscoring this experiment: the project team faced logistical issues which redirected the overarching project design, and the validation exercise–which was not the primary focus of the research–was affected and limited in turn. For future research, we encourage researchers to explore the potential of online data platforms for recruitment and hosting validation exercises. For example, in contrast to our study which took over 12 months to collect 30 responses, the research by [20] asked participants to draw maps of their neighbourhood on an online platform, and collected 1,086 responses in just three months (Dec 2022 –Feb 2023). Notwithstanding the potential efficiency savings of such a design, care and consideration must be given to evaluating whether online participants differ from the general resident population [21, 22]. As an improvement to the current design, we also recommend additional measures to establish the reliability and validity of the findings. Suggestions include redoing the exercise with residents after some time to determine test-retest reliability and more systematic integration of the validation exercise with analysis of interviews and other qualitative data; and implementing the exercise in a group setting such that respondents are encouraged to discuss their opinions and reach a collective decision.

A key motivation was to test the feasibility of the method: despite the aforementioned logistical challenges, this type of discrete choice experiment can be low-cost and quick to deploy: the creation of the research material itself took just one month and we have made our code available for other research. There is also significant potential to adopt the same or similar research design to establish the validity of different border detection approaches (Dean et al., 2019; Legewie and Schaeffer, 2016). Migration and migrant integration remains among the world’s most complex social and political challenges, and there is ample scope for further innovative, experimental research across a range of scales, including countries with distinct histories and patterns of domestic and international migration.

## Supporting information

S1 File Research materials and data. (ZIP)

S2 File Results and analysis notebook. (HTML)

S3 File Explanation of the Bayesian areal wombling method. (DOCX)
