# Supplementary material for: Using residents and experts to evaluate the validity of areal wombling for detecting social boundaries: A small-scale feasibility study
Source: PLoS One. 2024 Aug 26;19(8):e0305774. doi: 10.1371/journal.pone.0305774 (PMC11346722; doi:10.1371/journal.pone.0305774)
Supplement: S1 File — (ZIP) [file pone.0305774.s001.zip › materials/example choice.html]

# Which map contains borders that are more likely to be distinct boundaries between neighbouring communities?

Tip: Use the button pictured to open different background maps (e.g. street maps). Changes must be applied to both maps manually.

Tip: Use the plus button in the top left hand corner to zoom in and the minus button to zoom out.

Tip: Click and drag on the map to move the viewer.
