# Supplementary material for: Using residents and experts to evaluate the validity of areal wombling for detecting social boundaries: A small-scale feasibility study
Source: PLoS One. 2024 Aug 26;19(8):e0305774. doi: 10.1371/journal.pone.0305774 (PMC11346722; doi:10.1371/journal.pone.0305774)
Supplement: S1 File — (ZIP) [file pone.0305774.s001.zip › materials/Exercise script.docx]

**SF validation exercise**

Instructions [read the exact wording]:

We have already talked about some possible distinct boundaries between neighbouring communities in Rotherham West. As part of this research project, we've used a statistical model to predict borders that are likely to be distinct boundaries between communities.

The statistical model uses census data on the proportion of non-UK born residents in different areas to make its predictions (so settled vs. more recent population / migrants). We want your help to improve the statistical model.

Please take your time. You are not being tested.

If it's okay, can we start with a short preliminary exercise to get you used to our interactive maps?

I will show you three pairs of maps. Each map contains a different set of borders. Please explore the maps and choose the map in each pair that contains borders that are more likely to be distinct boundaries between neighbouring communities?
