# Supplementary material for: Using residents and experts to evaluate the validity of areal wombling for detecting social boundaries: A small-scale feasibility study
Source: PLoS One. 2024 Aug 26;19(8):e0305774. doi: 10.1371/journal.pone.0305774 (PMC11346722; doi:10.1371/journal.pone.0305774)
Supplement: S1 File — (ZIP) [file pone.0305774.s001.zip › materials/Preliminary-Exercise-clean.html]

Preliminary Exercise


# Preliminary Exercise

**Introduction**

As part of this research project, we’ve used a statistical model to predict borders that are likely to be distinct boundaries between communities. The statistical model uses census information on the proportion of non-UK born residents in different areas to make its predictions. We want your help to improve the statistical model.

If it’s okay, can we start with a short preliminary exercise to get you used to the interactive maps that we will be using? Please take your time.

**Instructions**: Look at the purple point in each map. Which map contains the point closest to Rotherham central station?

**Hints**:

- Use the button pictured to open the OpenStreetMap layer to enable you to view locations.
- Use the plus button in the top left hand corner to zoom in and the minus button to zoom out.
- Click and drag on the map to move the viewer.
