# Supplementary material for: Using residents and experts to evaluate the validity of areal wombling for detecting social boundaries: A small-scale feasibility study
Source: PLoS One. 2024 Aug 26;19(8):e0305774. doi: 10.1371/journal.pone.0305774 (PMC11346722; doi:10.1371/journal.pone.0305774)
Supplement: S2 File — (HTML) [file pone.0305774.s002.html]

Supplement S2: Results notebook


Code 

- Show All Code
- Hide All Code

# Supplement S2: Results notebook

#### 2023-04-19

Note: This report was generated in R markdown

# R code for setting up notebook

# Map data

```
# input -------------------------------------------------------------------

mapdataList <- readRDS('cleaned data/01 data for trial maps.rds')

# data wrangling ----------------------------------------------------------

mapdata <- bind_rows(
  a = mapdataList$a,
  b = mapdataList$b,
  c = mapdataList$c,
  .id = 'map'
)
```

```
# summaries ---------------------------------------------------------------


# other stats
mapdata %>% 
  as.data.frame() %>%
  group_by(map) %>%
  select(std_diff_phi:length, -xtile_rel) %>%
  summarise_if( 
    is.numeric, 
    list(mean = mean) 
#         `min/ max` = function(x){paste(min(x), '/' , max(x))})
  ) %>%
  table_me(
    digits = 2,
    col.name = c('map', 'mean phi (steepness, standardised)', 'mean rank of steepness (compared to region)', 'mean line length'),
    caption = 'Summary of border line characteristics (a = steepest map)'
    )
```

```
## Adding missing grouping variables: `map`
```

Summary of border line characteristics (a = steepest map)

| map | mean phi (steepness, standardised) | mean rank of steepness (compared to region) | mean line length |
| --- | --- | --- | --- |
| a | 3.01 | 97.42 | 726.7215 [m] |
| b | 1.21 | 78.75 | 627.9631 [m] |
| c | 0.20 | 28.36 | 662.6201 [m] |

```
## Rank is their rank in the entire Sheffield and Rotherham area
```

# Results data

```
# read the data -----------------------------------------------

result_df <-
  read_csv('cleaned data/makeFile03 cleaned experiment data.csv')


# 0. omit cases where the respondent did not pass initial prelim exercise
noPrelim <- c('RES_UK_R_23')

result_df <-
  result_df %>%
  filter(
    !(interview_id %in% noPrelim)
    )
```

The results data are kept in a csv table. Each row corresponds to one
map pair:

- interview\_id = id of the participant
- chronological\_id = id number in chronological order (1 = earliest
  interview)
- seenOrder = denotes whether a map pair was shown 1st, 2nd or
  3rd
- realPair = denotes which pair of maps was shown
  - 1: A vs B
  - 2: A vs C
  - 3: B vs C
- result = which map did participants pick (1 = left, 2 = right)
- mapA\_position = which map had the steepest borders (1 = left, 2 =
  right)
- duration1 = time in seconds taken to complete exercise (including
  preliminary task)
- interviewer = interview
- mode = whether interview was conducted face-to-face, online or via
  other means

```
result_df %>% 
  select(interview_id, chronological_id, seenOrder, 
         realPair, result, mapA_position, duration1, interviewer, mode) %>%
  head(9) %>%
  table_me(
    caption = 'Results example table'
  )
```

Results example table

| interview\_id | chronological\_id | seenOrder | realPair | result | mapA\_position | duration1 | interviewer | mode |
| --- | --- | --- | --- | --- | --- | --- | --- | --- |
| EXP\_UK\_R\_01 | 1 | 1 | 2 | 1 | 1 | 350 | Aneta | online |
| EXP\_UK\_R\_01 | 1 | 2 | 3 | 2 | 1 | 350 | Aneta | online |
| EXP\_UK\_R\_01 | 1 | 3 | 1 | 1 | 1 | 350 | Aneta | online |
| EXP\_UK\_R\_02 | 2 | 1 | 2 | 1 | 1 | 798 | Aneta | online |
| EXP\_UK\_R\_02 | 2 | 2 | 3 | 2 | 2 | 798 | Aneta | online |
| EXP\_UK\_R\_02 | 2 | 3 | 1 | 1 | 1 | 798 | Aneta | online |
| EXP\_UK\_R\_03 | 3 | 1 | 1 | 2 | 2 | 559 | Aneta | online |
| EXP\_UK\_R\_03 | 3 | 2 | 2 | 1 | 1 | 559 | Aneta | online |
| EXP\_UK\_R\_03 | 3 | 3 | 3 | 2 | 2 | 559 | Aneta | online |

# Agreement rate

```
# 1. analysis -------------------------------------------------------------

## Estimate the the aggrement rate 

nCases <- result_df$interview_id %>% unique %>% length ## number of participants

## Calculate the pvalue from a binomial dist with prob = 50% 
pVal_binom <-
  function(nSuccess, nTrials){
    1 - 2 * abs(0.5 - pbinom(nSuccess, nTrials, prob = 0.5))
    }

agreement_df <-
  result_df %>%
  mutate(`Map Pair` = realPair) %>%
  group_by(`Map Pair`) %>%
  summarise(
    n = n(),
    `n. agreed` = sum(mapA_position == result),
    `agreement rate` = `n. agreed` / n 
    )

agreement_df <-
  agreement_df %>%
  mutate(
    se = sqrt(0.25/ n), 
#    p.value = 2 * ( 1 - abs(agreeRate - 0.5) %>% pnorm(sd = se) ), #based on the normal distribution
    `p value` = pVal_binom(`n. agreed`, n) #based on the actual binomial dist 
    )

agreement_df %>% 
  table_me(
    caption = 'Table of agreement rates',
    digits = 4
  )
```

Table of agreement rates

| Map Pair | n | 14. agreed | agreement rate | se | p value |
| --- | --- | --- | --- | --- | --- |
| 1 | 30 | 29 | 0.9667 | 0.0913 | 0.0000 |
| 2 | 30 | 29 | 0.9667 | 0.0913 | 0.0000 |
| 3 | 30 | 18 | 0.6000 | 0.0913 | 0.2005 |

Map pairs legend:


- 1: A vs B
- 2: A vs C
- 3: B vs C

## Sequencing effects

```
# 2. check other stats -------------------------------------------------------

## seenorder = 

## sequence 
sequence_df <-
  result_df %>%
  group_by(seenOrder) %>%
  summarise(
    agreeN = sum(mapA_position == result),
    disagreeN = sum(mapA_position != result),
    agreeRate = agreeN / (agreeN +  disagreeN)
  )


sequence_df %>% 
  table_me(
    caption = 'Agreement rate by sequence',
    digits = 4
  )  ## i.e. does right answers change with the seen order (i.e. do we get higher agreement on the first maps seen)
```

Agreement rate by sequence

| seenOrder | agreeN | disagreeN | agreeRate |
| --- | --- | --- | --- |
| 1 | 27 | 3 | 0.9000 |
| 2 | 24 | 6 | 0.8000 |
| 3 | 25 | 5 | 0.8333 |

The above table shows the agreement rates maps pairs that are seen
first, second and third. We can use a Fischer exact test to check if the
agreement rate differs (not significant). We find no evidence of any
sequencing effects.

```
 sequence_df %>%
  select(agreeN, disagreeN) %>%
  fisher.test()
```

```
## 
##  Fisher's Exact Test for Count Data
## 
## data:  .
## p-value = 0.6673
## alternative hypothesis: two.sided
```

## Order

```
## order -------------------------------------------

result_df$mapA_position %>% 
  table %>% 
  table_me(
    col.names = c('Side (1 = left, 2 = right)', 'Freq'),
    caption = 'Side of steeper map'
  ) ## half the time the right map was on lhs
```

Side of steeper map

| Side (1 = left, 2 = right) | Freq |
| --- | --- |
| 1 | 44 |
| 2 | 46 |

```
## the data is random with respect to order 
order_df <- 
  result_df %>%
  group_by(mapA_position) %>%
  summarise(
    agreeN = sum(result == mapA_position),
    disagreeN = sum(result != mapA_position),
    agreeRate = agreeN / (agreeN + disagreeN)
    )## pretty much 50 - 50
##
order_df %>%
  table_me(
    col.names = c('side', 'n agree', 'n disagree', 'agreement rate'),
    caption = 'Agreement by order (i.e. whether steeper map is left or right) (1 = left, 2 = right)',
    digits = 3
  )
```

Agreement by order (i.e. whether steeper map is left or right) (1 =
left, 2 = right)

| side | n agree | n disagree | agreement rate |
| --- | --- | --- | --- |
| 1 | 36 | 8 | 0.818 |
| 2 | 40 | 6 | 0.870 |

The above tables check 1) whether the steeper map was more or less
likely to be on the left side and 2) whether the side affects agreement
rates. The Fischer exact tests the null hypothesis that there is no
difference in agreement rates (not statistically significant). We find
no evidence of any ordering effects.

```
order_df %>%
  select(agreeN, disagreeN) %>%
  fisher.test()
```

```
## 
##  Fisher's Exact Test for Count Data
## 
## data:  .
## p-value = 0.5693
## alternative hypothesis: true odds ratio is not equal to 1
## 95 percent confidence interval:
##  0.1756629 2.4746366
## sample estimates:
## odds ratio 
##  0.6779625
```

## Agreement over time

To check whether agreement rates rise over time, we split the sample
in half with one half consisting of the earliest interviews. We check
whether the agreement rate differs across the two halves. We find no
evidence of any changes in agreement rate over time.

```
## aggreement over time ----------------------------------------
## split the data into two halves 

timing_df <-
  result_df %>%
  mutate(
    splitTime = cut(chronological_id, 2)
    ) %>%
  group_by(splitTime) %>%
  summarise(
    agreeN = sum(mapA_position == result),
    disagreeN = sum(mapA_position != result),
    agreeRate = agreeN / (agreeN + disagreeN)
  )

timing_df %>%
  mutate(time = c('Earliest', 'Later')) %>%
  select(time, agreeN:agreeRate) %>%
  table_me(
    caption = 'Agreement rate over time',
    col.names = c('Timing', 'n agree', 'n disagree', 'agree rate'),
    digits = 4
    )
```

Agreement rate over time

| Timing | n agree | n disagree | agree rate |
| --- | --- | --- | --- |
| Earliest | 40 | 5 | 0.8889 |
| Later | 36 | 9 | 0.8000 |

```
timing_df %>% 
  select(agreeN, disagreeN) %>%
  fisher.test()
```

```
## 
##  Fisher's Exact Test for Count Data
## 
## data:  .
## p-value = 0.3837
## alternative hypothesis: true odds ratio is not equal to 1
## 95 percent confidence interval:
##  0.5374001 8.2749034
## sample estimates:
## odds ratio 
##   1.984821
```

```
## done. 
## No changes in agreement over time
```
