# Supplementary material for: Using residents and experts to evaluate the validity of areal wombling for detecting social boundaries: A small-scale feasibility study
Source: PLoS One. 2024 Aug 26;19(8):e0305774. doi: 10.1371/journal.pone.0305774 (PMC11346722; doi:10.1371/journal.pone.0305774)
Supplement: S3 File — (DOCX) [file pone.0305774.s003.docx]

One approach to calculating boundary values $\phi$is to use the absolute difference in proportions of a population between two adjacent areas. For example, let:

- $Y_{k}$ denote the total number of foreign-born residents in area (LSOA) $k; \mathrm{where} k=1,\ldots, n$
- $N_{k}$ denote the total number of residents in area $k$
- $p_{k}$ denote the proportion of residents in area $k$ who are foreign-born

For two adjacent areas ($k=1, k=2$ as examples), the absolute difference is:

$$\phi_{1,2}=|p_{k=1}-p_{k=2}|$$

The areal wombling algorithm used by [1] is based on a Bayesian spatial conditional autoregressive model. The model specifies the number of foreign-born residents $Y_{k}$ in LSOA $k$ as a function of the total residents $N_{k}$ and the probability of being foreign-born $p_{k}$.

$$Y_{k}\sim Binomial\left( N_{k}, p_{k} \right)$$

The logit transformation of $p_{k}$ is a linear function of an unknown parameter $\beta_{0}$ and a random effect $u_{k}$.

$$\ln\left( \frac{p_{k}}{1-p_{k}} \right)=\beta_{0}+u_{k}$$

The random effect $u_{k}$ is assumed to be spatially autocorrelated since the proportion of migrants in a zone $p_{k}$ is likely to be correlated with the proportion of migrants in neighbouring zones due to spillover effects and shared causal factors. A key component of the Bayesian spatial conditional autoregressive is the estimation of these random effects $u_{k}$ and a special spatial weights matrix driven by similarities between adjacent areas (e.g. to find adjacent areas that are dissimilar).

The full modelling approach and estimation are beyond the scope of this paper and are discussed in the supplementary materials of [1]. For purposes of estimating social boundaries, the boundary values between adjacent LSOAs $k=1$ and $k=2$ is equal to the absolute difference of the random effects predicted by the model:

$$\phi_{1,2}=|\tilde{u}_{k=1}= \tilde{u}_{k=2}|$$

For this study, we used the same code used in [1] (provided by the original paper authors and written in R).

**Bibliography**

1. Dean N, Dong G, Piekut A, Pryce G. Frontiers in Residential Segregation: Understanding Neighbourhood Boundaries and Their Impacts. Tijdschr Voor Econ En Soc Geogr. 2019;110: 271–288. doi:10.1111/tesg.12316
